# Sociodemographic factors and use of pain medication are associated with health-related quality of life: results from an adult community mental health service in Norway

**DOI:** 10.1007/s11136-023-03461-7

**Published:** 2023-06-20

**Authors:** Martin Schevik Lindberg, Martin Brattmyr, Jakob Lundqvist, Eirik Roos, Stian Solem, Odin Hjemdal, Audun Havnen

**Affiliations:** 1 Department of Psychology, Norwegian University of Science and Technology, NO-7491 Trondheim, Norway; 2 Health and Welfare, Trondheim Municipality, Trondheim, Norway; 3 Nidaros Community Mental Health Centre, Division of Psychiatry, St. Olav’s University Hospital, Trondheim, Norway

**Keywords:** Health-related quality of life, Mental health problems, EQ-5D-5L, Primary health services, Community health services

## Abstract

**Purpose:**

Health-related quality of life (HRQoL) is an important aspect of mental health outcomes. There are few studies on HRQoL in heterogeneous patient populations seeking help at community mental health services. The aims of the study were to compare how HRQoL, measured by the EuroQol five dimensions with five levels (EQ-5D-5L), was distributed compared to other samples from national and international studies, and to explore what factors are associated with HRQoL.

**Methods:**

In a cross-sectional study, 1379 Norwegian outpatients reported their HRQoL before starting treatment. Associations with demographic variables, job status, socio-economic status, and use of pain medication were examined using multiple regression analysis.

**Results:**

Most of the sample, 70% to 90%, reported problems with usual activities, pain/discomfort, and anxiety/depression; 30% to 65% reported that these problems were of a moderate to extreme degree. Forty percent reported problems with mobility, and about 20% reported problems with self-care. The sample’s HRQoL was considerably lower than the general population, and comparable to patient-groups from specialist mental health services. Originating from a developing country, lower level of education, lower yearly household income, being on sick leave or unemployed, and using pain medication were associated with lower HRQoL. Age, gender, and relationship status were not associated with HRQoL. This is the first study to simultaneously examine the unique contribution of these variables in one study.

**Conclusion:**

The most impacted domains of HRQoL were pain/discomfort, anxiety/depression, and usual activities. Lower HRQoL was associated with several socio-demographic factors and use of pain medication. These findings might have clinical implications and suggest that mental health professionals should routinely measure HRQoL in addition to symptom severity, to identify areas that should be targeted to improve HRQoL.

**Supplementary Information:**

The online version contains supplementary material available at 10.1007/s11136-023-03461-7.

## Introduction

Health-related quality of life (HRQoL) is commonly conceptualized as an individual’s perceptions of symptoms, physical-, social- and mental functioning, and well-being [1]. HRQoL is often used interchangeably with quality of life, and it has been argued that HRQoL should have a central role in mental health problem assessment [2]. This is supported by studies suggesting that HRQoL is a separate construct from symptoms [3], that quality of life might be an important predictor of treatment outcomes regardless of symptom severity [4], and that impaired quality of life persists after remission of depression symptoms [5]. In addition, HRQoL-outcome predicts return to work rates [6] and duration of sick leave [7].

The World Health Organization (WHO) has argued that increased access to mental health care may be achieved by providing more care outside mental health hospitals, such as primary health care and community mental health centers [8]. In Norway, community mental health services treat newly developed, less severe mental health problems, but also provide care for people with complex life challenges [9]. As HRQoL is an essential part of recovery and an indicator of quality in health services [10], strategies that contribute to the improvement of patients’ HRQoL should be prioritized. However, for HRQoL to be a useful concept in day-to-day practice and for decision makers, there is a need for knowledge about factors that influence patients’ HRQoL.

For patients in primary- and community mental health services, low HRQoL is associated with high symptom severity in major depressive disorder [11, 12] and anxiety disorders [13–15]. Previous studies have shown that symptoms account for 10% and 46% of the variance in HRQoL in patients with depression or anxiety in primary care, respectively (although a conceptual overlap between items in HRQoL-measures and symptom measures should be noted) [12, 14]. Other factors that influence HRQoL negatively are being on sick-leave [12] and being divorced or separated [13]. There are, however, conflicting findings regarding demographic variables such as age and gender [13, 14, 16], and socio-economic variables such as education and income [13, 17]. Moreover, for patients who receive treatment in specialist health services, pain is a predictor of worse HRQoL-outcomes [18–21], and research indicates ethnic inequalities regarding HRQoL in the general population, where being part of ethnic minorities is associated with lower HRQoL when compared to ethnic majorities [22–24].

The EuroQol five dimensions with five levels (EQ-5D-5L) [25, 26] is a widely used generic measures of HRQoL and is validated across a range of conditions and countries [27, 28]. The Norwegian EQ-5D-5L has been validated in the general population and for patients in specialist mental health services [29, 30]. However, there are no studies of EQ-5D-5L on samples from community mental health services. Thus, the first objective of this study was to investigate HRQoL measured by EQ-5D-5L in a community patient sample. We anticipated a degree of impairment in HRQoL worse than the general population, and more aligned with other clinical mental health samples.

The existing literature suggests an association between HRQoL and symptoms of anxiety and depression, being on sick leave, being single, ethnic background, and pain. The relationship between HRQoL and age, gender, and socio-economic variables remains unclear. Most of the previous research is based on disorder-specific studies, while patients in routine care are usually heterogeneous [31]. Thus, there appears to be a gap between what is known about patients’ HRQoL from earlier studies, and what characterizes HRQoL in patients treated in routine community mental health care. The second objective was therefore to explore what patient characteristics are associated with HRQoL in routine community mental health services. Consistent with previous findings, we expected higher severity of symptoms of anxiety and depression, being out of work, and living without a partner to be negatively associated with HRQoL. Based on mixed previous findings, we also wanted to investigate if income level, education level, originating from a developing country, and higher levels of pain are negatively associated with HRQoL. To our knowledge, the relative contribution of these variables in explaining HRQoL has not been investigated before.

## Method

### Participants and procedure

In an observational cross-sectional study, data were sampled from treatment-seeking adults at an outpatient community mental health service in the municipality of a large Norwegian city. All responses were registered before treatment-start in the period September 2020 – May 2022. Participants gave their consent and responses through a web-based portal. Of 1858 patients, 1379 consented to participate in the study (74% response rate). There were no exclusion criteria for participation, but people who needed home-based treatment were excluded because they receive help in other health care units in the municipality.

The target group of the mental health service is a mix of (1) people with recently developed mild to moderate mental health problems, (2) people with mental health problems in addition to more complex life-challenges, and (3) people with concurrent addiction and mental health problems. The first group comprised 66% of the total sample and was self-referred. This group was not diagnosed according to the International Classification of Diseases 10 (ICD-10) but was assessed over telephone by health professionals with a bachelor’s degree (such as nurses and social workers with education in mental health) or clinical psychologists. The second group, 24% of the sample, was mainly referred by general practitioners or health professionals in the specialist mental health services. Half of the second group was registered with either a P-diagnosis from the International Classification of Primary Care (ICPC-2) [32] or an F-diagnosis from ICD-10. The largest diagnosis-groups were anxiety-disorders (38%), depressive disorders (36%), personality disorders (19%), attention-deficit disorder (13%), addiction-disorders (10%), obsessive–compulsive disorder (9%), post-traumatic stress disorder (6%), and autism-spectrum disorder (5%). Fifty-seven percent had two or more diagnoses. In the third group, 10% of the sample, 72% contacted the service without a referral, while 28% were referred.

The sample’s mean age was 36.0 years (12.7), ranging from 18 to 82. Sixty-five percent of the sample identified themselves as women, 33.5% as men, 1.0% as non-binary, and 0.5% as transgendered. The 1.5% who identified as non-binary and transgendered were not part of regression analyses due to their low frequency. Sample characteristics are described in Table 1.

**Table 1:** Sample characteristics and tests of differences between sexes

	Total (*n* = 1363)	Women (*n* = 901)	Men (*n* = 462)	*d* or *V*
EQ-5D-5L				
EQ-5D values	0.559 (0.247)	0.559 (0.244)	0.562 (0.253)	0.03
VAS (Health 0–100)	52.9 (19.6)	52.6 (19.1)	54.1 (20.3)	0.11
PHQ-9	13.7 (5.8)	13.7 (5.6)	13.5 (6.1)	0.00
GAD-7	11.4 (4.7)	11.6 (4.6)	10.8 (4.7)	0.16^*^
Age	36.0 (12.7)	35.9 (12.4)	36.2 (12.2)	0.02
		%		
Age groups				0.05
18–29	38.4	40.4	34.8	
30–39	28.4	26.8	31.4	
40–49	16.1	16.5	16.1	
50–59	10.8	10.2	12.1	
60 +	5.9	6.1	5.5	
Relationship status				0.05
Not in a relationship	58.2	57.0	60.8	
Job-/income status				0.05
Ordinary income	55.9	57.6	52.5	
Sick leave	26.7	26.0	29.1	
Unemployed	17.4	16.4	18.4	
Use of pain medication	13.4	14.4	10.3	0.07^**^
Country of origin				0.02
Developing country	6.8	6.3	8.0	
Education				0.20^***^
Not finished primary school	0.6	0.7	0.5	
Primary school	10.5	9.6	11.6	
High school	35.8	32.5	42.3	
Vocational school	9.1	8.7	9.9	
University bachelors	20.9	22.4	18.4	
University master’s level and higher	23.1	26.1	17.3	
Yearly household income				0.01
Less than 250,000	24.9	23.8	26.8	
250,100 to 450,000	24.5	24.5	23.8	
450,100 to 750,000	23.6	24.5	22.4	
750,100 to 1,000,000	13.0	13.3	12.7	
More than 1,000,000	14.0	13.9	14.3	

*HRQoL* Health related quality of life, *VAS* Visual Analog Scale, *GAD-7* Generalized Anxiety Disorder-7, *PHQ-9* Patient Health Questionnaire-9

* *p* < 0.05, ** *p* < 0.01, *** *p* < 0.001

### Measures

The following background variables were self-reported: age; gender; relationship status (dichotomized as in a relationship or not in a relationship); job status (grouped in three categories: 1) having ordinary income which included full time work, part-time work and being a student; 2) being on sick leave; and 3) being unemployed); regular use of pain medication (using medication or not using medication); country of origin using the United Nation’s definition (from a developing country or not from a developing country) [33]; education level; and yearly household income. The median yearly household income in Norway was approximately 550,000 NOK in 2020 [34].

EQ-5D-5L [25] was used to assess HRQoL. It comprises a visual analogue scale (VAS) which asks the respondents to rate their overall health on a scale ranging from 0 (worst health) to 100 (best health) and a descriptive system based on five dimensions: mobility, self-care, usual activities, pain/discomfort, and anxiety/depression. The five dimensions have five levels of responses (1–5), where a higher score indicates more severe problems. A combination of responses in these five dimensions gives a *health state profile*. Health state profiles can be used to describe a patient’s level of problems in the five dimensions and can also be converted to *EQ-5D values* consisting of a single score number with three decimals. The EQ-5D values are produced from country specific weights based on preference studies sampled from the general population [25, 35]. As country-specific weights for Norway do not exist, a “crosswalk” technique recommended by the Norwegian Medicines Agency was used [36], which involves mapping the EQ-5D-5L health state profiles on to the existing value-set from UK EQ-5D-3L [37]. The EQ-5D values can produce negative values, but scores usually range from 0, which represents being dead, to 1 which represents full health. Norwegian norm data has been published [29], and a systematic review indicates good psychometric properties [27]. As the five dimensions measure five different domains, a high internal consistency was not expected (Cronbach’s α = 0.74).

The Patient Health Questionnaire-9 (PHQ-9) [38] and Generalized Anxiety Disorder-7 (GAD-7) [39] were used as measures of symptom severity of depression and anxiety, respectively. PHQ-9 has a sum-score ranging 0–27, and GAD-7 has a sum-score ranging 0–21, with higher total scores indicating more severe symptoms. A PHQ-9 sum-score ≥ 10 has a sensitivity of 88% and a specificity of 88% for major depressive disorder [38], while a GAD-7 sum-score of ≥ 10 has a sensitivity of 89% and a specificity of 82% for generalized anxiety disorder [39]. PHQ-9 and GAD-7 scores are commonly interpreted as following: 0–5 mild, 6–10 moderate, 11–15 moderate/severe, and 15 + severe. PHQ-9’s and GAD-7’s psychometric properties are well examined both internationally and in Norway [40–42]. The items of PHQ-9 and GAD-7 demonstrated acceptable levels of internal consistency (Cronbach’s α = 0.85; Cronbach’s α = 0.84).

### Statistical analyses

Multiple regression analyses were used to examine relationships between HRQoL and the independent variables. The VAS and the EQ-5D values were used as dependent variables in separate multiple hierarchical regression analyses. The regression analyses were structured in five steps with the following variables added in each step. Step 1: Age, gender, and country of origin. Step 2: Yearly household income and education level. Step 3: Job status. Step 4: Relationship status. Step 5: Use of pain medication. For the model with VAS as dependent variable, an additional analysis was conducted controlling for symptoms of anxiety and depression, together with the aforementioned covariates. Due to the conceptual overlap between EQ-5D-5L's dimension anxiety/depression and GAD-7 and PHQ-9, symptoms of anxiety and depression were not controlled for in the analysis using EQ-5D values as outcome. Multicollinearity was not a problem between any of the analyzed variables, with VIF values ranging 1.04–1.96.

Previous studies indicate that having more than one mental health disorder or having concurrent mental health problems and problems with addiction are associated with lower HRQoL [43–45]. Sensitivity analyses were conducted to check if either were associated with lower HRQoL, or if their inclusion in the analyses changed the effects of the other variables. In addition, there is a high prevalence of pain in older adults, and many use pain medication regularly [50]. Sensitivity analyses were carried out to check whether the part of the sample over 60 years old contributed to skewed results in the regression analyses.

For the total sample, the rate of missing responses was low for the variables age, EQ-5D values, VAS, PHQ-9, and GAD-7 (0.1–5%). The rate of missing for gender, originating from a developing country, education, yearly household income, and job status was 7%. Relationship-status and use of pain medication were not answered by 14% of the respondents. The explanation for the high rates of missing responses on the two latter variables were that they were included later in the data collection than the other variables. This issue exacerbated high baseline rates of missing responses for the participants with concurrent addiction and mental health problems (135 patients, 10% of the total sample). The rate of their missing responses ranged 0–1.5% on age, EQ-5D value, VAS, PHQ-9, and GAD-7, while the rate of missing on gender, originating from a developing country, education, yearly household income, job status, relationship status, and use of pain medication ranged 40–50%. A test of whether the data were missing completely at random showed that they were not. With multiple imputation [46], 200 imputed datasets were analyzed in multiple regression analyses with VAS and EQ-5D values as outcomes. All data handling and statistical analyses were done in the statistical environment of *R* [47], and the *mice* package was used for multiple imputation [48]. Standardized regression coefficients for the imputed datasets were computed with the method *Standardization before Pooling* [49].

## Results

### Descriptive statistics

Background variables, EQ-5D values, VAS, GAD-7, and PHQ-9 stratified by men and women are shown in Table 1. The mean score on the EQ-5D values and VAS were 0.559 (0.247) and 52.9 (19.6), respectively. The mean score on PHQ-9 was 13.7 (5.8) and the mean score on GAD-7 was 11.4 (4.7). Both scores represent moderate/severe symptoms of anxiety and depression [38, 39]. Seventy-eight percent had a sum score ≥ 10 on either GAD-7 or PHQ-9, while 53% had a sum score ≥ 10 on both GAD-7 and PHQ-9. Women used more pain medication, had higher education, and had higher scores on GAD-7 compared to men. The gender categories non-binary and transgendered, comprised fourteen persons and two persons, respectively. The two categories combined had a mean score of 0.503 (0.260) on the EQ-5D values, VAS scores were 55.3 (22.2), GAD-7 scores were 13.4 (4.5), and PHQ-9 scores were 16.4 (6.2). The non-binary/transgendered participants scored approximately equal to men and women on VAS scores, but they had lower mean EQ values and higher mean scores of GAD-7 and PHQ-9.

The distribution of EQ-5D-5L item-responses was stratified by age groups of 18–29, 30–39, 40–49, 50–59, and ≥ 60, and separate for women and men. Forty-two percent of women and 38% of men had problems with mobility, and 21% of women and 18% of men had problems with self-care. Almost 80% reported problems in the dimension of usual activities. A larger proportion of women (81%) reported problems in the dimension of pain/discomfort than men (75%). Women (89%) and men (92%) reported similar degrees of problems in the dimension of anxiety/depression. For detailed information on the distribution of item-responses of EQ-5D-5L, see Supplementary Tables 1 and 2.

### Comparison of VAS and mean item scores with other studies

The participants’ mean score on VAS was lower than the general population in Norway [29], and about the same as a Norwegian sample of mixed anxiety and depression from the specialist health services [30]. The VAS was lower compared to a British study with depressed patients and Dutch patients with personality disorders [28], but higher compared to a Spanish study with depressed patients [11]. In the dimension anxiety/depression, the current sample reported more problems than the British sample with depression, fewer problems than the Spanish sample with depression, and similar to the sample with personality disorder. The current sample reported more problems in the dimension of pain/discomfort compared to the sample with personality disorder and the British sample with depression, but less problems than the Spanish sample with depression. Regarding usual activities, the current sample reported more problems than the depression sample, but less problems than the sample with personality disorder and the Spanish sample with depression. Differences in EQ-5D-5L scores are shown in Table 2.

**Table 2:** Comparing EQ-5D-5L VAS and mean item scores with other studies by *t*-tests

	Current study^a^	Garrat et al., 2021^b^	Janssen et al., 2013^c^	Janssen et al., 2013^d^	Bilbao et al., 2021^e^	Difference *p* < 0.001^f^
Sample	Community mental health care	General population	Personality disorder	Depression	Depression	
Country	Norway	Norway	Netherlands	UK	Spain	
Scores						
VAS	52.9 (19.6)	77.9 (18.3)	59.0 (18.0)	62.0 (21.0)	46.9 (21.9)	b,c,d > a > e
Mobility	1.7 (0.9)	1.3 (0.7)	1.2 (0.6)	1.6 (0.9)	2.0 (1.1)	b,c < a < e
Self-care	1.3 (0.7)	1.1 (0.4)	1.1 (0.3)	1.3 (0.7)	1.6 (0.9)	b,c < a < e
Usual activities	2.4 (1.0)	1.4 (0.8)	2.5 (1.1)	1.9 (1.0)	2.5 (1.2)	b,d < a
Pain/discomfort	2.4 (1.0)	1.9 (0.9)	2.0 (1.0)	2.1 (1.1)	2.7 (1.2)	b,c < a < e
Anxiety/depression	2.9 (1.1)	1.5 (0.8)	2.9 (1.1)	2.6 (1.1)	3.1 (1.2)	b,d < a < e

^a^Norwegian sample from community mental health services

^b^General population from Norway [29]

^c^Dutch sample with personality disorder from specialist mental health services [28]

^d^British sample with major depressive disorder from specialist mental health services [28]

^e^Spanish sample with major depressive disorder from hospitals and primary care [11]

^f^Comparisons between the current study and the other respective studies; only significant results are shown (*p* < 0.001)

### Variables associated with HRQoL

In the regression model using EQ-5D values as outcome-variable (Table 3), the following steps contributed significantly to the variance explained: Demographic variables, socio-economic status, job status, and use of pain medication. The final step in the model explained 18% (adjusted *R*^*2*^). In the fully adjusted model, the following variables were associated with lower EQ-5D values: originating from a developing country, lower education level, lower yearly household income, being unemployed, being on sick leave, and use of pain medication.

**Table 3:** Regression analysis predicting HRQoL (EQ-5D values)

Step	Variable	Adjusted *R*^*2*^	*R*^*2*^ change		*F* change
1	Demographic variables	0.00	0.03		10.0^***^
2	Socio-economic status	0.11	0.08		54.4^***^
3	Job status	0.14	0.03		24.5^***^
4	Not in a relationship	0.14	0.00		2.1
5	Use of pain medication	0.18	0.04		44.7^***^

Final step		*Estimates*^c^	*Lower CI*	*Upper CI*	*p*
Age		− 0.04	− 0.09	0.02	0.208
Gender		− 0.01	− 0.12	0.10	0.901
Developing country of origin		− 0.46	− 0.67	− 0.25	< 0.001
Education level		0.17	0.11	0.22	< 0.001
Household income		0.14	0.07	0.21	< 0.001
Unemployed^a^		− 0.46	− 0.62	− 0.30	< 0.001
Being on sick leave^b^		− 0.31	− 0.43	− 0.18	< 0.001
Not in a relationship		0.10	− 0.03	0.23	0.139
Use of pain medication		− 0.56	− 0.73	− 0.40	< 0.001

*p < 0.05, ** *p* < 0.01, *** *p* < 0.001

*HRQoL* Health related quality of life, *CI* 95% Confidence Interval

^a^Job status category “Ordinary income” was used as reference level

^b^Job status category “Ordinary income” was used as reference level

^c^Estimates were standardized

For the model with VAS as outcome (Table 4), the following steps contributed to the total variance: Socio-economic status, job status, and use of pain medication. The fully adjusted model explained 11% (adjusted *R*^*2*^) of the variance in VAS. The following variables were significantly associated with lower VAS: lower education level, lower yearly household income, being unemployed, being on sick leave, and use of pain medication.

**Table 4:** Regression analysis predicting HRQoL (VAS)

Step	Variable	Adjusted *R*^*2*^	*R*^*2*^ change		*F* change
1	Demographic variables	0.00	0.00		1.0
2	Socio-economic status	0.05	0.05		27.1^***^
3	Job status	0.08	0.03		27.9^***^
4	Not in a relationship	0.08	0.00		0.0
5	Use of pain medication	0.11	0.03		28.6^***^

Final step		*Estimates*^c^	*Lower CI*	*Upper CI*	*p*
Age		0.00	− 0.05	0.06	0.873
Gender		− 0.10	− 0.21	0.01	0.086
Developing country of origin		− 0.02	− 0.25	0.20	0.835
Education level		0.11	0.05	0.17	< 0.001
Household income		0.12	0.05	0.19	0.001
Unemployed^a^		− 0.32	− 0.49	− 0.16	< 0.001
Being on sick leave^b^		− 0.46	− 0.59	− 0.34	< 0.001
Not in a relationship		0.01	− 0.12	0.14	0.874
Use of pain medication		− 0.46	− 0.63	− 0.29	< 0.001

*p < 0.05, ^**^ *p* < 0.01, ^***^ *p* < 0.001

*HRQoL* Health related quality of life, *VAS* Visual Analog Scale, *CI* 95% Confidence Interval

^a^Job status category “Ordinary income” was used as reference level

^b^Job status category “Ordinary income” was used as reference level

^c^Estimates were standardized

The analysis with VAS as outcome was repeated controlling for symptoms of anxiety (measured with GAD-7) and depression (measured with PHQ-9). In this analysis, education level and income were no longer significantly associated with VAS. Being unemployed, being on sick leave and use of pain medication, as well as depression symptoms, were significant variables. The fully adjusted model explained 33% (adjusted *R*^*2*^) of the variance in VAS. As sensitivity analyses, we performed complete cases analyses in addition to the regression analyses with imputed datasets, with equal results between the two analyses.

Diagnoses were registered for a sub-group of the total sample (*n* = 196), where half of them had two or more diagnoses. To investigate if comorbidity was associated with lower HRQoL, an additional sensitivity analysis was conducted (two or more diagnoses vs. one or no diagnoses registered). Having two or more diagnoses was associated with lower EQ-5D values (*p* < 0.001, 95% CI [− 0.72–− 0.32]) and VAS (*p* < 0.001, 95% CI [− 0.61–− 0.19]). However, controlling for this variable did not change the effects of the remaining variables in the analyses. Additional regression analyses were conducted to check if controlling for concurrent mental health problems and addiction reduced the effects of the other variables. In the regression analyses, a dummy variable of having concurrent mental health and addiction was included. Having concurrent mental health problems and addiction was associated with lower EQ-5D values (*p* = 0.007, 95% CI [− 0.43–− 0.07]), but not with the VAS (*p* = 0.922, 95% CI [− 0.18–0.20]). The results of the other variables remained unchanged.

To investigate whether the part of the sample who were over 60 years old (5.9%) contributed to skewed results in the regression analyses, all participants over the age of 60 were removed. The results of the regression analyses were identical as the analyses with participants over 60 years old included, in both complete-cases and imputed analyses.

## Discussion

The two main objectives of this study were to investigate HRQoL measured by EQ-5D-5L in patients seeking treatment in the community mental health services, and to explore what factors are associated with HRQoL. The sample was characterized by low HRQoL across a broad range of areas, with several associated characteristics. Specifically, originating from a developing country, having lower education levels and lower yearly household income, being on sick leave or unemployed, and use of pain medication were associated with lower HRQoL. When controlling for symptoms of anxiety and depression in the regression analysis with VAS as the outcome, being on sick leave or unemployed, use of pain medication, and symptoms of depression remained associated with lower self-reported health.

Assessed on the VAS, the participants rated their health as lower than the general population in Norway [29], and about equal to a Norwegian sample from the specialist health services [30]. The VAS was lower than a British sample of depressed patients and Dutch patients with personality disorders [28], but higher compared to a Spanish sample of depressed patients [11]. Measured on EQ-5D-5L’s five dimensions, the current sample reported considerably lower HRQoL than the general population, and similar HRQoL as disorder-specific patient groups from specialist mental health services [11, 28]. Previous findings suggest that cultural differences might influence the response pattern of the EQ-5D-3L [50]. Although the current sample had lower HRQoL than the samples from Great Britain and the Netherlands, the scores were not markedly different. However, the Spanish sample had distinctly lower HRQoL compared to all the three other patient samples and were lower on all measures except for usual activities. This might be related to cultural factors, as previous studies have noted that the Spanish society is more collectivistic and has higher scores on uncertainty avoidance in relation to countries in northern Europe [50]. Additionally, fewer people with mental health problems were in contact with health services compared to countries in northern Europe such as Denmark, Sweden, the Netherlands, and Great Britain [51], and they had higher use of psychotropic medication [52].

The second objective was to explore the relationship between different patient characteristics and HRQoL represented by EQ-5D values and VAS. Sick leave and being unemployed were associated with lower HRQoL, which is in accordance with previous findings [12, 17, 30]. Due to the cross-sectional data, we cannot infer any causal direction of this relationship, as being away from work may cause low HRQoL and vice versa. Previous findings have indicated that low HRQoL is associated with longer durations of sick leave [7]. An implication of this finding is that mental health professionals should be aware that the combination of being on sick leave and reduced HRQoL might represent a risk for longer term absence from work. Important goals for treatment should therefore include strategies that support employment or returning to work.

Lower education level was associated with lower HRQoL and adds to the body of conflicting findings regarding education and HRQoL. Two studies from primary care found that lower education was negatively associated with physical aspects but not mental aspects of HRQoL [13, 15]. One study found no difference in HRQoL between education levels [12]. Another reported that more years of education were associated with worse mental aspects of HRQoL [17]. Having a lower yearly household income was also associated with lower HRQoL. This is in line with findings from primary care [15] and mixed samples from primary care and specialized health care [17]. Nevertheless, the current findings that education and income are associated with HRQoL are in line with research on socio-economic factors and HRQoL in other groups, such as persons with chronic diseases [53], and the general population in Norway [54] and the USA [55]. As lower income is associated with financial debt, which in turn is associated with mental health problems [56], health professionals should routinely assess patients’ financial situation. Increased financial stability and measures directed at continuing education and training could work as routes to better HRQoL.

Use of pain medication was associated with lower HRQoL, which corroborates previous studies where pain was associated with worse HRQoL-outcomes [19, 21]. As the EQ-5D-5L includes a question about pain/discomfort, there is a potentially conceptual overlap between the variables in the analysis. However, the VIF-analysis indicated no multicollinearity, and the use of pain medication was also associated with VAS. This suggests that use of pain medication is associated with HRQoL, both when reported as EQ-5D values and VAS. This finding is important, as the use of pain medication may be an indication of how the individual relates to pain and may influence a person’s perceived HRQoL. Health professionals should routinely assess whether patients use pain medication and assess whether they have problems related to pain in general. Pain management might be preferred to medication, to improve both self-efficacy and HRQoL. If the pain is severe or chronic, referral to specialized treatment should be considered.

In the general population, originating from a developing country or being part of an ethnic minority are associated with lower HRQoL [22, 23]. This relationship was found for EQ-5D values but not VAS. This may indicate that persons originating from countries with major societal differences compared to Norway could experience greater difficulties when adapting to the Norwegian culture and society. However, originating from a developing country is not necessarily a cause of low HRQoL, as there could be multiple third variables in play. In addition, as argued by Nesterko [22], immigrants represent quite heterogenous groups. Thus, it may be beneficial to control for specific countries of origin to identify whether certain ethnic minorities might be more vulnerable to having impaired HRQoL than others.

We found no association between age and HRQoL, which is in line with some previous research [16, 30]. However, older age has also been related to lower HRQoL in physical aspects, and lower age has been related to mental problems [13, 14]. The lack of association between age and HRQoL may be due to the age-distribution in our sample, with very few respondents in the higher age groups.

Except for a higher proportion of women reporting problems with pain/discomfort compared to men, problems in the remaining domains were reported to a similar degree. These results are consistent with several other studies [13, 14, 16], but not a Norwegian study [30], in which the authors suggested that women have higher rates of sick leave in general, whereas we did not identify gender differences regarding sick leave and HRQoL. Nor was civil status associated with HRQoL, which was somewhat unexpected given that previous research shows that being separated or divorced is associated with lower HRQoL [13]. However, we dichotomized this variable as being in a relationship or not, which may have influenced the results.

Since the EQ-5D-5L measures anxiety and depression as a separate domain, the EQ-5D values also reflect mental health problems. However, to investigate if self-reported health, assessed on the VAS, could be influenced by symptoms of anxiety and depression, we conducted an additional analysis controlling for this. As expected, symptoms of anxiety and depression contributed to a substantial amount of the explained variance in VAS. Symptoms of depression, but not symptoms of anxiety, were significantly associated with lower self-reported health. This is consistent with results from a study of patients with anxiety disorders in primary care, where major depression disorder was the unique predictor of mental aspects of HRQoL [13]. However, the results from the present study contradict those of two other studies from primary care, where anxiety and depression were equally strong predictors of HRQoL [14, 15]. A possible explanation may be that a large proportion of the current sample had concurrent symptoms of anxiety and depression, which makes it hard to disentangle the unique contribution of anxiety and depression, respectively. To improve patients’ subjective experience of health, health professionals should use interventions that can reduce symptoms of anxiety and depression.

Additional sensitivity analyses were conducted to test whether comorbidity of mental health diagnoses or concurrent mental health and addiction affected the results of the regression analyses. The results were in line with previous research showing that psychiatric comorbidity and comorbid addiction both were associated with lower HRQOL, but they did not, however, change the effects of the other variables.

A strength of the current study is the large sample size and the inclusion of a broad range of potential predictors of HRQoL. However, some limitations should be noted. Only parts of the sample were diagnosed, and the duration of the patient’s mental health problems was unknown. This reduces the validity of comparisons between the present study and studies with disorder-specific samples. Furthermore, due to the study’s cross-sectional design, no causal inferences between variables can be made. The low number of individuals reporting gender as non-binary and transgender excluded parts of the sample from analysis. There were high rates of missing information on background variables for patients with concurrent mental health and addiction problems. Although multiple imputation is a robust technique for handling missing responses, this might reduce the representativeness for this group of patients. Another potential limitation to the study was that the period of data-collection included periods with societal restrictions because of the COVID-19 pandemic. Even though levels of mental disorders in Norway during the COVID-19 pandemic were reported to be stable compared to pre-pandemic levels [57], other studies have suggested a minimal to small increase in symptoms of anxiety and depression in high income countries [58]. This might reduce the generalizability of the findings to time periods without similar societal restrictions.

## Conclusion

The results indicate that people seeking help in community mental health services have considerably lower HRQoL than the general population and approximately equal levels as patients who receive treatment in specialist mental health services. Previous findings indicate that being out of work, living without a partner, income level, education level, originating from a developing country, and higher levels of pain are associated with HRQoL, but conflicting findings exist. This study is the first to examine the unique contribution of all these variables in the same study. In this sample, lower HRQoL was associated with originating from a developing country, having lower education and lower household income, being on sick leave or unemployed, and using pain medication.

### Supplementary Information

Below is the link to the electronic supplementary material.

Supplementary file1 (PDF 126 KB)

## Data Availability

Data are available from the corresponding author upon reasonable request.
