# Correction: Sociodemographic factors and use of pain medication are associated with health-related quality of life: results from an adult community mental health service in Norway

**DOI:** 10.1007/s11136-023-03549-0

**Published:** 2023-11-04

**Authors:** Martin Schevik Lindberg, Martin Brattmyr, Jakob Lundqvist, Eirik Roos, Stian Solem, Odin Hjemdal, Audun Havnen

**Affiliations:** 1 Department of Psychology, Norwegian University of Science and Technology, NO-7491 Trondheim, Norway; 2 Health and Welfare, Trondheim Municipality, Trondheim, Norway; 3 Nidaros Community Mental Health Centre, Division of Psychiatry, St. Olav’s University Hospital, Trondheim, Norway

**Correction: Quality of Life Research (2023) 32:3135–3145** **https://doi.org/10.1007/s11136-023-03461-7**

In Table 4 of the original published article, the data in the row “Developing country of origin”, column “Upper CI” should be 0.20, whereas it was mistakenly published as – 0.20.

The correct version of Table 4 with the correct entry is provided below.

**Table 4:** Regression analysis predicting HRQoL (VAS)

Step	Variable	Adjusted *R*^2^	*R*^2^ change	*F* change
1	Demographic variables	0.00	0.00	1.0
2	Socio-economic status	0.05	0.05	27.1***
3	Job status	0.08	0.03	27.9***
4	Not in a relationship	0.08	0.00	0.0
5	Use of pain medication	0.11	0.03	28.6***

Final step	*Estimates*^c^	*Lower CI*	*Upper CI*	*p*
Age	0.00	− 0.05	0.06	0.873
Gender	− 0.10	− 0.21	0.01	0.086
Developing country of origin	− 0.02	− 0.25	0.20	0.835
Education level	0.11	0.05	0.17	< 0.001
Household income	0.12	0.05	0.19	0.001
Unemployed^a^	− 0.32	− 0.49	− 0.16	< 0.001
Being on sick leave^b^	− 0.46	− 0.59	− 0.34	< 0.001
Not in a relationship	0.01	− 0.12	0.14	0.874
Use of pain medication	− 0.46	− 0.63	− 0.29	< 0.001

*p < 0.05, **p < 0.01, ***p < 0.001

*HRQoL* Health related quality of life, *VAS* Visual Analog Scale, *CI* 95% Confidence Interval

^a^Job status category “Ordinary income” was used as reference level

^b^Job status category “Ordinary income” was used as reference level

^c^Estimates were standardized

The original article has been corrected.

