# Supplementary material for: Sociodemographic factors and use of pain medication are associated with health-related quality of life: results from an adult community mental health service in Norway
Source: Qual Life Res. 2023 Jun 20;32(11):3135–45. doi: 10.1007/s11136-023-03461-7 (PMC10522514; doi:10.1007/s11136-023-03461-7)
Supplement: Supplementary file 1 — Supplementary file1 (PDF 126 KB) [file 11136_2023_3461_MOESM1_ESM.pdf]

Sociodemographic factors and use of pain medication are associated with health-related quality of life: results from an adult community mental health service in Norway

Submitted to the journal *Quality of Life Research*

Martin Schevik Lindberg[1,2], Martin Brattmyr[1], Jakob Lundqvist[1], Eirik Roos[2], Stian Solem[1], Odin Hjemdal[1] & Audun Havnen[1,3]

Corresponding author:

Martin Schevik Lindberg

Norwegian University of Science and Technology

NTNU, Department of Psychology, NO-7491, Trondheim, Norway

martin.s.lindberg@ntnu.no

Supplemental Table 1. Distribution of EQ-5D scores for women by age groups.

EQ-5D-5L dimensions	Proportion of responses in every dimension					All women
	18-29	30-39	40-49	50-59	>=60	
	%	%	%	%	%	%
Mobility						
No problems	55.3	62.0	61.2	56.5	51.1	58.1
Slight problems	27.3	24.0	23.3	21.2	20.0	24.6
Moderate problems	14.3	9.2	12.4	14.1	20.0	12.9
Severe problems	3.1	4.8	3.1	7.1	6.7	4.1
Unable to do	0.0	0.0	0.0	1.1	2.2	0.3
Proportion reporting problems	44.7	38.0	38.8	43.5	48.9	41.9
Self-care						
No problems	74.8	83.3	79.7	81.2	82.6	79.2
Slight problems	18.3	12.3	13.3	14.1	15.2	15.1
Moderate problems	5.4	3.1	3.9	3.5	2.2	4.1
Severe problems	1.5	0.9	3.1	1.2	82.6	1.5
Unable to do	0.0	0.4	0.0	0.0	15.2	0.1
Proportion reporting problems	25.2	16.7	20.3	18.8	17.4	20.8
Usual activities						
No problems	18.3	21.0	17.2	30.6	33.3	21.0
Slight problems	41.9	44.1	46.1	34.1	31.1	41.6
Moderate problems	25.8	24.9	24.2	20.0	20.0	24.5
Severe problems	13.4	10.0	11.7	12.9	15.6	12.3
Unable to do	0.6	0.0	0.8	2.4	0.0	0.6
Proportion reporting problems	81.7	79.0	82.8	69.4	66.7	79.1
Pain/discomfort						
No problems	22.6	18.3	16.5	10.6	13.1	18.6
Slight problems	39.3	46.3	36.7	43.5	23.9	40.6
Moderate problems	26.9	24.9	29.7	27.1	34.8	27.1
Severe problems	8.7	10.0	14.8	16.5	15.2	11.2
Extreme	2.5	0.5	2.3	2.3	13.0	2.5
Proportion reporting problems	77.4	81.7	83.5	89.4	86.9	81.4
Anxiety/depression						
No problems	9.3	12.2	12.5	16.7	4.4	11.2
Slight problems	24.5	32.8	31.5	29.7	28.9	28.7
Moderate problems	35.7	34.0	38.6	28.5	20.0	34.2
Severe problems	25.5	17.5	15.8	19.1	40.0	21.7
Extreme	5.0	3.5	1.6	6.0	6.7	4.2
Proportion reporting problems	90.7	87.8	87.5	83.3	95.6	88.8

Supplemental Table 2. Distribution of EQ-5D scores for men by age groups.

EQ-5D-5L dimensions	Proportion of responses in every dimension					All men
	18-29	30-39	40-49	50-59	>=60	
	%	%	%	%	%	%
Mobility						
No problems	62.2	61.2	61.8	57.5	57.9	61.4
Slight problems	22.4	21.6	16.2	21.3	21.0	20.8
Moderate problems	10.5	13.4	13.2	14.9	5.3	12.1
Severe problems	4.2	2.2	5.9	4.2	5.3	3.9
Unable to do	0.7	1.6	2.9	2.1	10.5	1.9
Proportion reporting problems	37.8	38.8	38.2	42.5	42.1	38.6
Self-care						
No problems	77.5	83.5	72.1	85.1	79.0	79.6
Slight problems	15.5	12.0	17.6	4.3	15.8	13.4
Moderate problems	4.2	4.5	8.8	8.5	0.0	5.3
Severe problems	2.8	0.0	1.5	2.1	5.2	1.5
Unable to do	0.0	0.0	0.0	0.0	0.0	0.2
Proportion reporting problems	22.5	16.5	27.9	14.9	21.0	20.4
Usual activities						
No problems	24.5	19.6	26.5	25.5	31.6	23.7
Slight problems	30.8	39.1	26.5	27.7	31.6	32.7
Moderate problems	34.3	21.8	26.5	34.0	21.0	28.1
Severe problems	9.7	16.5	17.7	10.6	15.8	13.5
Unable to do	0.7	3.0	3.0	2.2	0.0	2.0
Proportion reporting problems	75.5	80.4	73.5	74.5	68.4	76.3
Pain/discomfort						
No problems	29.6	26.3	17.7	21.3	21.0	25.5
Slight problems	43.7	45.9	51.4	38.3	26.3	43.9
Moderate problems	21.1	19.6	19.1	21.3	31.6	20.6
Severe problems	4.2	6.8	7.4	17.0	5.3	7.3
Extreme	1.4	1.4	4.4	2.1	15.8	2.7
Proportion reporting problems	70.4	73.6	82.3	78.7	79.0	74.5
Anxiety/depression						
No problems	6.3	10.5	13.2	2.1	0.0	8.3
Slight problems	27.5	19.6	30.9	31.9	16.7	25.3
Moderate problems	31.0	39.9	33.8	40.4	55.6	36.5
Severe problems	28.2	23.3	14.7	23.4	22.2	23.6
Extreme	7.0	6.7	7.4	2.2	5.5	6.3
Proportion reporting problems	93.7	89.5	86.8	97.9	100.0	91.7
